# Books, &c., Received

**Published:** 1850-10-01

**Authors:** 


### Miscellaneous Notices.

### BOOKS, &c., RECEIVED FOR REVIEW.

*A Selection of the Papers and Prize Essays on Subjects connected with Insanity, read before the Society for Improving the Condition of the Insane.* London: 1850.

THIS work has disappointed us. We have gone carefully through the prize essays, and, with a few exceptions, we regret to say that we see but little to commend. The volume opens with an essay "On Restraint and Coercion," by Dr. Haslam, said to be read April 3, 1833. How is this? The Society, we are informed in the preface, was not constituted until 1843. Surely Dr. Haslam's essay is quite out of place. With every respect for the name of that eminent physician, we cannot but observe that these are not the times for a Society, professing to have for its object the improvement of the condition of the insane, to put so prominently forward a defence of restraint and coercion. If Dr. Haslam's paper was not a *prize* essay, it has no right to be printed at all in the volume. If the Society was instituted in 1843, how, we ask, could the essay of Dr. Haslam be read before its members in 1833, *ten* years previously to its organization? The volume contains, also, essays on "Crime and Insanity," on the "Increase of Insanity," a case by Sir A. Morison, M.D., two essays on "Puerperal Insanity," on the "Pathology of the Brain," on "Lucid Intervals," on the "Medical Treatment of Mental Diseases," and on the "Moral Management of the Insane." The three last essays are those most deserving of our commendation. Dr. L. Robertson enters fully both into the medical and moral treatment of insanity. The former essay must have entailed upon him great research, and shows extensive knowledge of the literature of the subject.

*On Corpulence, or Excess of Fat in the Human Body, &c.* By T. K. Chambers, M.D., Fell. Roy. Coll. Phys., and Gulstonian Lecturer for 1850. 1 vol. Longman, &c.

As it is our wish to confine our reviews to works which relate specially to the subject of psychological medicine, we very much regret that it is not in our power to give a full analysis of Dr. Chambers' able and very interesting treatise on corpulence. The substance of the work was contained in a course of lectures delivered by the author at the College of Physicians, as Gulstonian lecturer. This is, we believe, the first attempt to treat the subject of corpulence in the spirit of modern science. Dr. Chambers' work ought to be read and studied both in and out of the profession. The work does him great credit. He has quite exhausted the subject.

*A Lecture introductory to the Course of Surgery, delivered at the Massachusetts College, Boston.* By H. J. Bigelow, M.D. Boston: 1850.

This is an excellent introductory lecture, and speaks well for the ability, knowledge, and zeal of its author.

*The Law Magazine, for August,*

Contains an article on the plea of lunacy, at variance with the views propounded in the observations in the present number on Pate's trial. We received the Number after our article was in type.

*Konesipathy; or, the Cure of Diseases by Specific Active and Passive Movements.* By Augustus Georgii. 1850.

In this treatise, the author considers the subject of medical gymnastics in all its important details. We agree with the writer that the advantages of a regular, scientific, and systematic code of gymnastics are not sufficiently appreciated by the public and profession. The work before us enters very fully into the subject, and deserves perusal. The author is disposed to overrate the curative efficacy of muscular exercise; nevertheless his observations (which are free from all empiricism) are entitled to careful consideration. The work is well written, and embodies the literature of the subject.

*The Medical Guide.* By Richard Reece, M.D. 16th edition. Edited by his Son, Henry Reece. 1850. Longman.

This volume contains a vast fund of useful information. The instructions for the cure and prevention of disease are conveyed in intelligible language. Mr. Henry Reece has carefully edited his father's work, and has embodied in it the most recent improved views of the treatment of disease.

*The Accommodation of the Eye to Distance.* By W. C. Wallace, M.D. New York, 1850.

A Scientific Essay on Optics, with appropriate engravings, illustrative of the author's visionary views. The wood-cuts are not equal to the letter-press of the work. However, the remarks of the author are clear; and acting upon the principle which he enunciates, and accommodating our own editorial eyes to a distance, and glancing across the Atlantic, we should say that Dr. Wallace was a good anatomist, and sound physiologist. We trust the worthy Doctor will excuse a little psychological pleasantry.

*Proceedings of the Fifth Annual Meeting of the Association of Medical Superintendents of American Institutions for the Insane.*

We have to thank Dr. S. Kirkbride of Philadelphia for an early copy of this pamphlet. We can truly say that the Americans are really "going a-head" in psychological matters. The society promises great things. We shall watch its proceedings with interest, and give our readers a faithful record of its progress.

*The London Medical Examiner.* (The Series.)

The Editor of this Journal handles his pen with ability. There is always something of interest and value in this periodical.

*The London Journal of Medicine* (regularly).

This is one of the most able and scientific of our monthly journals. It is conducted with great judgment, and the articles are always upon points of practical importance. Some of the most able physicians and surgeons in the metropolis rank among its contributors.

*A Plea of Humanity in Behalf of Medical Education; an Annual Address, delivered before the New York State Medical Society.* By Alexander H. Stevens, M.D., LL.D. Fourth Edition. New York. 1850.

A valuable contribution to American medical literature. We are much pleased with the high moral tone of this essay. Its general perusal would tend to elevate the character of the profession in the estimation of the public.

*On Diseases of Menstruation and Ovarian Inflammation.* By Edward J. Tilt, M.D. London. Churchill.

In this able treatise, the author discusses the question "Should marriage be sanctioned, and when?" This is a subject of deep interest to the psychologist, and as he has often to deal with cases of insanity, complicated with uterine derangement and ovarian disease, every work which throws light on these subjects should be carefully studied. The derangements of the mind are so often complicated with disordered uterine function, that it becomes absolutely necessary for the physician to fully acquaint himself with the best works on this subject. We can recommend Dr. Tilt's treatise to the notice of our readers.

*On Juvenile Crime: a Letter to the Town Council, &c., of Liverpool.* By Edward Rushton, Esq., Stipendiary Magistrate.

In our next Number this pamphlet will be noticed at some length.

*Observations on Chronic Hydrocephalus, Acquired, Sanguineous, and Congenital; with an account of three cases in which the head was punctured, and an examination of the effects of that operation.* By Francis Battersby, M.B., F.R.C.S.I., &c. Edinburgh. 1850.

We have seldom read a more able and erudite treatise on any given medical subject than the one before us. Dr. Battersby has, in addition to his own original views, made himself master of every work, pamphlet, and essay, on the subject of hydrocephalus, and has embodied the product of his immense labours in his own treatise. We very much regret that our limited space will not admit of our giving a full analysis of this very excellent and learned production.

*On a much less Painful and more Scientific Method of Extracting the Teeth.* By Henry Gilbert, Esq., M.R.C.S. L. & L.A.S. Second Edition.

This little volume has received the commendation of the principal portion of the medical press of the country, and it deserves the praise bestowed upon it. Every attempt to apply the principles of science to dentistry is entitled to warm commendation.

